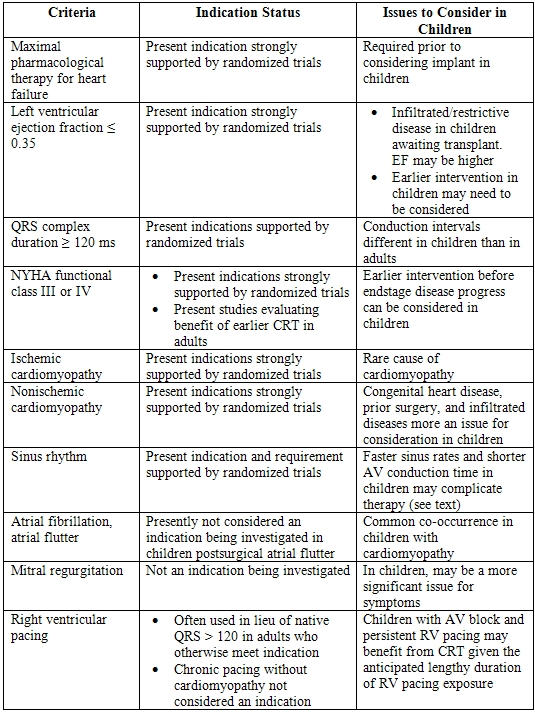# An Approach to Overcoming Specific Difficulties with Cardiac Resynchronization Therapy in Children

**Published:** 2008-05-01

**Authors:** Megan M Constans, Samuel J Asirvatham

**Affiliations:** Division of Cardiovascular Diseases, Department of Internal Medicine, Mayo Clinic, Rochester, Minnesota

**Keywords:** cardiac resynchronization therapy, children

## Introduction

Cardiac resynchronization therapy (CRT) is now a well established treatment modality for adult patients with drug refractory symptomatic congestive heart failure. Multiple large-scale studies have clearly demonstrated reduction in heart failure-related morbidity with CRT [1-4]. More recently, a likely independent mortality benefit with CRT has also been shown [5]. Improvement in quality of life, decrease in left ventricular diastolic dimension, improved objective assessment of exercise tolerance, and decreased heart failure-related hospitalizations have all been well demonstrated in the adult population. Whether or not these benefits occur in younger patients is not clear [6]. Since none of the major trials for CRT have included children, much is unknown about the specifics of indication, difficulties with implant, and efficacy in the pediatric population [7].

More recently, small case series have shown the potential for benefit for CRT in patients with congenital heart disease including those with a systemic right ventricle and single ventricle physiology. Whether long-term benefit for extending CRT therapy to children with congenital heart disease exists is unclear.

There are several challenges confronting the implanter when considering CRT therapy in children, and these are summarized in Table 1.

Despite these limitations in our present knowledge as to how indications, implant, and optimization of CRT devices should be amended in children, evidence is accumulating that suggests at least a similar benefit in children exists, as has been been demonstrated in adults.

In children with left ventricular failure and left bundle-branch block, acute left ventricular hemodynamics has been shown to improve with biventricular stimulation [8]. In longer-term studies, CRT has also been demonstrated to improve effort tolerance and oxygen consumption [9-14]. Isolated reports have shown an average increase in ejection fraction of 14-18% and typically correlated with a concurrent decrease in QRS duration of 20-40 ms. Improvement (decrease) in left ventricular end diastolic diameter with effective resynchronization of up to 6 mm has been reported [15]. Improvement in heart failure symptoms and New York Heart Association class has also been shown anecdotally to occur in children as has been well established in adults [14,16,17]. Given this rudimentary but existing evidence of CRT benefit for children with refractory heart failure symptoms, the implanting physician needs to be familiar with the idiosyncrasies of pediatric CRT. Not only must the implanter be cognizant of the indications, techniques for optimization, and problems with implant seen in adults, but understand specifically the unique challenges of placing optimally a left ventricular pacing lead in children.

In this article, we discuss commonly encountered causes for difficulty in implanting CRT devices in children with and without congenital heart disease. We suggest potential solutions and an approach in solving such difficulties. We further present guidelines for understanding differences in interpreting the standard indications for CRT implantation and optimization of CRT devices in adults to children [18].

## Difficulties with Implanting Left Ventricular Leads in Children

Implanting left ventricular leads in children is generally more challenging than in adults. The reasons for this, as expected, are largely a result of the smaller size of the vessels and cardiac chambers as well as the increased risk of cardiac perforation as with most pediatric cardiac procedures. Beyond this, however, there are specific challenges that are addressed in this section involving challenges often unique to the pediatric population.

### Cannulating the Coronary Sinus

Entering the coronary sinus in children requires an accurate understanding of the standard fluoroscopic views and important anatomic landmarks in these views. Vagaries of the coronary ostium itself in the young and the immediately adjacent atrial myocardium is important to appreciate, particularly when selecting sheaths and manipulating the pacing lead. A prominent thebesian valve at the ostium of the coronary sinus likely occurs more frequently in this age group as well.

#### Fluoroscopy

**Figure 1:**
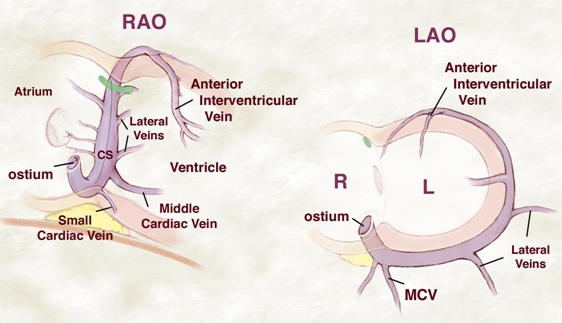
***Coronary Sinus and Ventricular Veins:*** It is essential to appreciate the normal course and anatomic relations of the coronary sinus in two standard fluoroscopic views. Note, the course from posterior to anterior and right to left in the LAO projection, whereas in the RAO projection the coronary sinus main body is neither anterior nor posterior and stays in a single plane. Ventricular branches are anterior to the main body of the CS and when cannulated should be immediately recognized.

Because the ventricular apex is to the left of the body, the fluoroscopic views must take this into consideration to obtain anatomic accuracy. The use of the left anterior oblique (LAO) and right anterior oblique (RAO) projections is highly recommended and can be used to avoid common complications (Figure 1). In the LAO view, the coronary sinus extends from the right atrium to the operator's right of the screen. This characteristic movement to our right (left atrium) is an important indicator that the coronary sinus had been entered. It should be remembered, however, that when there is severe cardiac enlargement, the heart may be rotated, and the right ventricular apex may be very "leftward" appearing. By looking at the orthogonal view (RAO), it will be easy to differentiate between deep ventricular engagement and that of the coronary sinus. In the RAO view, the coronary sinus catheter or pacing lead advances neither anteriorly (ventricle) nor posteriorly (atrium) but appears to come right out at the operator. A common and usually successful maneuver is to take a soft bidirectionally deflectable catheter that has been placed through a guiding sheath. The catheter is first placed in the right ventricle posteriorly. Strong counterclockwise torque is then applied to force the catheter towards the intraventricular septum. Now, holding this same torque, the catheter is gently pulled back until a characteristic to-and-fro movement is noted, suggesting cannulation of the coronary sinus. This maneuver is done in the RAO projection, and as soon as the change in movement is noted, the fluoroscopic camera can be moved to the LAO view to be certain that the deflectable catheter is advancing to the operator's right (patient's left) as is typical of the coronary sinus. Once entered, the guiding sheath is then moved over the catheter to gain access to the proximal portion of the coronary sinus, preferably about 1-2 cm distal to the coronary sinus ostium.

Performing this maneuver in every case in the standard fluoroscopic projections will significantly enhance the ease in which this important first step for CRT delivery is performed. As seen in Figure 1, at times, the ostium and proximal portion of the coronary sinus can be fairly tortuous, but again, noting that the catheter when being advanced does in fact go to the operator's right in the LAO projection and neither anteriorly or posteriorly in the RAO projection and using gentle force, the CS can be safely negotiated. An additional fluoroscopic landmark is that in the RAO projection at the junction of the diaphragm and cardiac silhouette a lucency just posterior to the coronary sinus called the epicardial "fat pad" can be appreciated. This is an approximate landmark for the operator to know as to when to expect entering the coronary sinus when pulling back the catheter torqued onto the septum as explained above.

#### Prominent Thebesian Valve

Rarely, a completely occlusive imperforate thebesian valve may preclude standard cannulation of the coronary sinus. Even with near occlusive valves, typically the inferior-ventricular quadrant of the coronary sinus ostium will not be covered. This is one of the reasons why the maneuver described above starting from the ventricle as inferiorly as possible and pulling back to the coronary sinus is more likely to be successful in the difficult case. In the rare patient with a near occlusive valve, gentle force with a blunt catheter being certain that one is in the plane of the coronary sinus and using intracardiac or transesophageal ultrasound guidance is successful. In very rare circumstances, radiofrequency energy posteriorly applied (to avoid AV nodal damage) can be used to create a controlled perforation of the thebesian valve and allow entrance of the coronary sinus.

#### The Subeustachian Pouch

**Figure 2:**
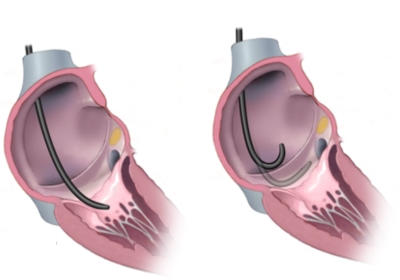
A subeustachian pouch when present and the coronary sinus occur in the same anatomic plane between the eustachian ridge and the tricuspid annulus. When a guiding sheath or catheter is pulled back with counterclockwise torque from the ventricle, the catheter may fall into the pouch. When it appears to the operator that the coronary sinus has not been cannulated in that plane, failing to recognize the presence of the pouch may result in the operator pulling further back behind the eustachian ridge when cannulating the CS would then become impossible.

An important anatomical fact should be appreciated when implanting left ventricular leads in children. The atrial myocardium between the eustachian ridge and the tricuspid valve immediately adjacent to the coronary sinus ostium is often aneurysmal or pouch-like in young children. This subeustachian pouch (Figure 2) can make placement of a guiding sheath in an appropriate plane to cannulate the CS difficult. When pulling back the catheter or sheath with counterclockwise torque being applied, the sheath will appear to suddenly jump. At this point, gentle injection of contrast dye will show swirling or stagnation in the subeustachian pouch. The operator may mistakenly think that further counterclockwise torque is required, and then the guiding sheath or catheter will move behind the eustachian ridge, making entering the coronary sinus impossible. Understanding in the RAO projection when the subeustachian region has been entered can make negotiating the CS easier. When this difficulty is noted, it is best to use guiding sheaths that do not require stability on the inferior atrial wall such as relatively straight guiding sheaths or sheaths with very large secondary curvatures that "balance" against the lateral right atrial wall (straight Attain™ or Worley™ sheaths).

### Difficulty with Advancing within the CS

Once the coronary sinus has been entered and the guiding sheath stabilized in its proximal portion, the next task that may be difficult in children is advancing the pacing lead within the coronary sinus to a ventricular vein that drains the lateral wall of the left ventricle.

#### Coronary Sinus Dissection

Particularly in the very young, the coronary vein can be quite friable, and even a relatively moderate manipulation can result in coronary venous dissection. Although this is well tolerated by patients, once dissection occurs, further manipulation in the coronary sinus or placing the lead may be precluded. Perhaps the most important method to avoid coronary dissection in addition to applying very gentle force is to never advance an oversized sheath (inner diameter of the sheath larger than the outer diameter of the wire or guiding catheter within the sheath) in tandem into the coronary sinus. Once a guiding catheter has been placed relatively deep into the coronary vein, it should be pulled back as the sheath is being advanced. Similarly, when advancing the pacing lead once the guide wire has advanced to a vein of interest, when trying to advance the lead, the wire should be gently pulled back as the lead is advanced. After advancing about a centimeter, the wire can then be re-advanced into the ventricular vein of interest and the maneuver repeated until the lead is in the required position. This maneuver (pulling back on wire while pushing the lead) is particularly important to execute when negotiating tortuous vessels or at the point of branching in the ventricular veins and tributaries.

#### Sub-selection

A common reason for difficulty in advancing the pacing lead or causing venous damage is inadvertent sub-selection of an atrial or ventricular branch of the coronary sinus. If the operator is unfamiliar with the expected course of the coronary sinus in the RAO and LAO projections, an atrial branch may have been entered after engaging the coronary sinus. With continued forward force application or further counterclockwise torque, the lead/catheter will not only fail to advance but dissection may occur. Similarly, soon after engaging the coronory sinus, a posterior ventricular vein may have been entered. Again, without familiarity with this possibility, the operator may think that the coronary sinus has not been entered and the lead/catheter is in the right ventricle and pull out of the vein when simply advancing the lead or sheath over the catheter to allow lead placement may have given a satisfactory result.

#### Valves

**Figure 3:**
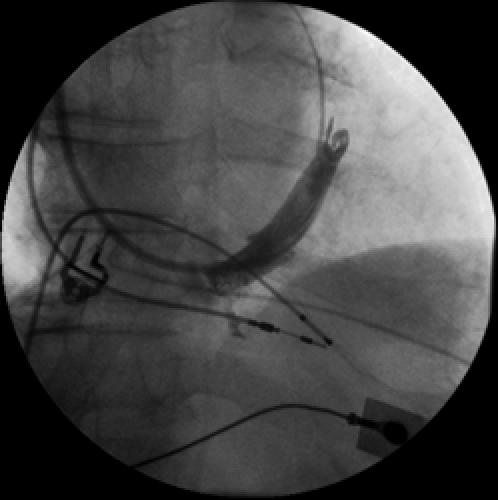
***Near Occlusive Valve:*** LAO CS angiogram demonstrating a near occlusive valve about 3 cm from the coronary sinus. This is a frequent site to find the valve of Vieussens.

Just as the thebesian valve "guards" the ostium of the coronary sinus, various valves may be present, particularly in children, throughout the course of the coronary sinus including the junction with the great cardiac vein and at the ostia of the various ventricular venous branches. The most consistent of these valves is the so-called Vieussens valve located at the ostium of the posterolateral vein, typically at the same location of the vein of Marshall (oblique atrial vein) approximately 2-3 cm from the coronary sinus ostium in children. This valve, if small, does not cause much difficulty once a wire has crossed the valve and entered either the posterolateral ventricular vein or great cardiac vein. When nearly circumferential, however, this may preclude entering the posterolateral vein, a typical target for left ventricular lead implantation (Figure 3). When this occurs, either a more anterior lateral or a more posterior vein can be entered. A tributary of one of these veins draining the lateral wall can be used for satisfactory left ventricular pacing [10].

#### Collaterals

**Figure 4:**
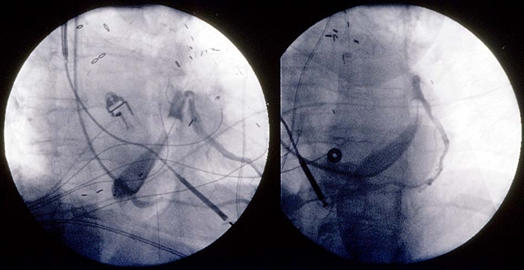
***Posterolateral and Middle Cardiac Vein:*** RAO (left panel) and LAO (right panel) projections showing a typical anastomotic vein between the posterior and anterior circulation that traverses the lateral wall. Placing a lead via either of these veins using the anastomosis is entirely identical to pacing the lateral wall through a vein through a direct lateral vein. When difficulty occurs in negotiating a lateral vein, using an anterior or posterior vein and the linking anastomosis should be considered.

The ideal target to place the left ventricular pacing lead in most patients is the midportion of the left ventricular free wall. There is typically a lateral cardiac vein draining this location into either the great cardiac vein or coronary sinus [18]. Sometimes, however, the takeoff of this vein may be very tortuous, or the vein itself may be too small to safely cannulate. It is important for operators to be aware of extensive collaterals that exist between the anterior, posterior, and lateral venous circulation on the free wall of the left ventricle. These collaterals are usually of sufficient size in children to take some of today's smaller diameter over-the-wire left ventricular pacing leads. The typical maneuver required is to first cannulate the posterior or anterior cardiac vein with the lead. With the lead placed in this vein, then using a soft-tip guide wire (Whisper™) the collateral is entered. Once entered, gently pulling back on the guide wire while advancing the lead will sub-select the collateral. In the LAO projection, the lead should be seen to be on the lateral wall. In addition, when pacing from a true lateral wall location, a QS wave (negative deflection) in lead I of the electrocardiogram will be noted (see below). A dictum worth remembering for operators is that the left ventricular free wall site and not the vein draining it directly is the target for pacing the lead (Figure 4).

## Left Ventricular Lead Implantation in Congenital Heart Disease

Anecdotal and in relatively small case series [19,20], CRT has been shown to be beneficial in improving cardiac function and quality of life in various congenital heart diseases. The implanter, however, must have an accurate knowledge of the relevant cardiac anatomy, location of the coronary sinus, site of emptying of the ostium of the coronary sinus, and whether or not right-to-left shunting exists that would preclude endovascular implantation. In this section, we will review common causes of difficulty and a general approach for implanting leads in patients with congenital heart disease.

### Persistent Left Superior Vena Cava

**Figure 5:**
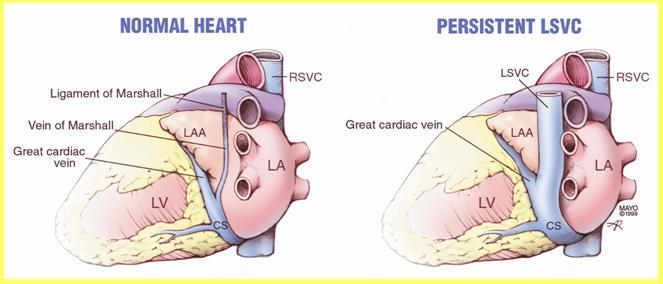
Illustration of the consistent anatomic position of the left superior vena cava between the left atrial appendage and the left-sided pulmonary veins. The oblique vein of Marshall can be used to pace the left atrium.

Several congenital anomalies may have an associated persistent left superior vena cava. However, this anomaly often exists as a separate entity. The right superior vena cava may exist in addition to the persistent left superior vena cava, and one or more anastomotic veins between the vena cavae may be present. During initial experience with CRT, operators assumed that using the left superior vena cava would be easier for accessing the coronary sinus and a ventricular vein since this structure drains into the coronary sinus (Figure 5). However, lead stability is not optimal in these cases since the coronary sinus and ventricular veins are often grossly enlarged. The author's preference when the right superior vena cava cannot be accessed is to cannulate the left subclavian vein and place a lead into the main body of the coronary sinus via the left SVC. Once the lead reaches this location, counterclockwise torque is applied so that the guide wires, lead, and sheath will travel anterolaterally to the junction of the left SVC with the coronary sinus into a more anterior vein. An anterolateral or lateral branch of an anterior vein is then sub-selected and the left ventricular pacing lead placed.

When the left superior vena cava is not entirely patent, it may remain as a smaller oblique vein of Marshall (Figure 5). In certain children requiring biventricular pacing, to obtain ideal left atrial-left ventricular synchrony, a left atrial pacing lead also requires to be placed. For those patients, the coronary sinus is entered in a manner described above. However, a deflectable catheter is advanced about 2-3 cm into the coronary sinus and then a small posterior deflection is made and the catheter further rotated in a counterclockwise direction, observing catheter movement in the RAO projection. Atrial vein engagement is noted by posterior movement in the RAO projection and the recording of large atrial electrogram from the pacing lead or mapping catheter.

In the setting of more complex congenital heart disease, the left superior vena cava may offer the best option to access both ventricles (endocardially and via the coronary venous system). Angiography from the vein and from the left superior vena cava is sometimes useful to understand the best target, guiding sheath, and catheter torque to obtain optimal results [21].

### Where is the Coronary Sinus?

A critical question that must be asked by the implanter and thoroughly researched with review of prior admitting data is where the coronary sinus drains in the patient being considered for CRT and with congenital heart disease [17]. For example, in a patient with an older-type of Fontan procedure, wherein the right atrial appendage is connected to the right ventricular outflow tract, the coronary sinus will drain normally into the right atrium. Thus, even in a patient with tricuspid atresia, ventricular (specifically left ventricular) pacing can be effected via an endocardial route utilizing the coronary sinus [12].

Exact surgical details and recent imaging data are essential before proceeding. In patients, for example, with AV canal defects, the closure of the septum primum defect and/or placement of a prosthetic tricuspid valve may be done in such a way that the coronary sinus actually drains into the right ventricle, distal to the tricuspid valve placement. For such patients, the catheter that is being used in an attempt to engage the coronary sinus must first access the ventricle and then appropriately maneuvered to cannulate the coronary sinus. Right ventricular as well as right atrial angiography may be useful, for even when the coronary sinus has been excluded from the right-sided structures accessible through the vena cavae, large thebesian veins may be draining into the basal right ventricle that can be cannulated and a left ventricular pacing lead placed either directly or via anastomoses to the coronary veins.

### Where is the Apex and Which Ventricle is Anterior?

In addition to clearly understanding the detailed anatomy of a patient's congenital defect, the operator should be aware of where the apex is located and the relative positions of the right and left ventricles.

**Figure 6:**
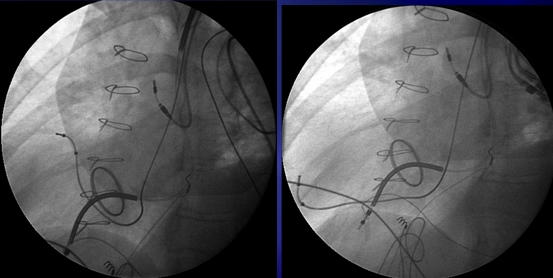
Fluoroscopic images (right panel RAO, left panel LAO) of CRT-D implantation in a patient with dextrocardia and L-TGA. Knowledge of where the coronary sinus opens and the relative position of the right and left ventricles are essential to successful implant in such cases [22,23].

Knowledge of where the apex is located will allow the operator to adjust the fluoroscopic views accordingly. For example, in a patient with dextrocardia, the RAO angle is lined up along the axis of the apex and will in effect be used like the LAO projection to know when the coronary sinus is cannulated and whether the ventricular free wall is being accessed for pacing. The orthogonal view, in this case the LAO projection, will be used similarly to the RAO projection in the normal heart to follow the typical course of the coronary sinus and ventricular veins. If a patient has mesocardia, then the AP becomes the effective LAO view (Figure 6).

Once the preimplant knowledge of the apex and coronary sinus drainage is known, it can be very useful to know the relative positions of the two ventricles. If the right ventricle is anterior, generally, coronary sinus lead placement is not distinctly more difficult than with normal anatomy. However, when the right ventricle is the posterior ventricle, careful venography, possible coronary arteriography to view the venous phase, and placement of a lead in the right ventricle prior to attempting coronary sinus cannulation can be useful.

### L-TGA

In patients with L-TGA and morphological right ventricular failure, CRT may be beneficial [24,25]. The coronary venous anatomy is unusual in these patients [25]. The coronary sinus itself goes with the atrium, that is, the left atrium is normally located in these patients, and the coronary sinus follows the usual radiographic course. However, the ventricular veins are more similar to the typical right ventricular veins. Thus, a distinct lateral, anterolateral, or posterolateral vein is usually not present. The veins, however, may be large enough to place a pacing lead, otherwise the middle cardiac or anterior intraventricular vein are cannulated and lateral branches used for pacing. The morphological left ventricle (systemic venous circulation) drains primarily through the septal veins (anterior intraventricular and middle cardiac vein) and large thebesian veins that directly empty into the right atrium.

### ICD Leads in the Coronary Sinus and Ventricular Veins

**Figure 7:**
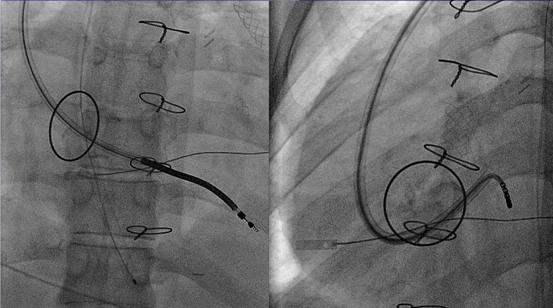
RAO (left panel), LAO (right panel) fluoroscopic images of placing a defibrillator lead in the coronary venous system in a patient with a prosthetic tricuspid valve. Children in whom the ICD lead should not be placed across the tricuspid valve can have the ICD lead placed in a posterior ventricular vein (see text for details).

In patients with a prosthetic tricuspid valve, it may not be possible (mechanical prosthesis) or inadvisable (bioprosthesis) to cross the valve to place an ICD lead in the right ventricle. In these patients, using similar techniques (Figure 7) described above to cannulate and place a lead in the coronary veins, a guiding sheath can be used to sub-select a posterior ventricular vein. Because of the relatively shorter length of the ICD leads, standard guiding sheaths may need to be cut to allow passage of the ICD lead into the vein of interest. Both tined as well as active fixation leads have been used in this situation. When an active fixation mechanism is used, care must be taken in using the RAO projection to see that the screw is being deployed towards the ventricular myocardium and not in the free wall of the middle or posterior cardiac veins.

### Epicardial Placement of the Left Ventricular Lead

Patients with intracardiac shunts have a contraindication to placement of an endocardial system. The majority of experience with epicardial pacing leads for left ventricular stimulation comes from pediatric experience. Epicardial leads are frequently used in this situation because of the smaller ventricular veins in infants and small children. The use of epicardial leads is documented to have shorter longevity and higher incidence of associated exit block in comparison to endocardial steroid-eluting leads. Recently, however, the creation of steroid-eluting epicardial leads and longer duration follow-up have shown that acceptable results can be obtained with these pacing systems [26-28]. The steroid-eluting tip reduces inflammation, decreasing exit block, and keeps electrical thresholds in an acceptable range, an important consideration given the long duration of lead requirement when placed in younger children. Most successful cases of CRT in congenital heart disease in infants have been with an epicardial pacing system in Europe and the United States [22,29]. A suggestion from these studies [22] is that in children weighing less than 55 pounds, an epicardial biventricular pacing lead should be considered as a first option because of the small veins. The author's preference, however, is to attempt endocardial pacing as the first option and resort to epicardial implant when this fails.

### Bridge to Cardiac Transplant

In recent years, CRT along with assist devices has been used as a bridge to heart transplant. Patients on heart transplant waiting lists have established criteria for CRT [27]. Some patients [23] have been taken off the transplant list based on unmistakable improvement in ejection fraction and clinical status following CRT [30].

## Selection Criteria: Challenges for the Pediatric Population

Even in the adult population, intensive study is presently being done to understand which exceptions to the standard criteria for recommending CRT therapy should be made. In the pediatric population, however, these issues are paramount to consider. Very few pediatric patients, both in the literature and in practice, meet the adult criteria for CRT, yet have benefited from this form of therapy [31].

Present guidelines include having patients in New York Heart Association (NYHA) class III-IV, normal sinus rhythm, electrical evidence of ventricular dyssynchrony with QRS duration greater than 120 ms, and left ventricular ejection fraction less than 35% [31,32]. Further, there is evidence that when standard pacemakers are placed in young patients, they tend to require pacing for many years, and CRT may offset RV pacing-induced cardiomyopathy [33]. The present indications for CRT, future considerations, and specific issues related to children are summarized in Table 2 and elaborated on below.

Electrical dyssynchrony, as evidenced by bundle-branch block or wide QRS duration, is likely associated with mechanical dyssynchrony. This is not, however, a consistent relationship. This discordance (between QRS duration and likelihood of CRT benefit) is more pronounced in children since their normal QRS duration and intraventricular conduction time tend to be smaller [18,31,34,35]. Thus, children with highly symptomatic heart failure despite optimal medical therapy should not be precluded from consideration of CRT based on QRS duration alone. Therapy must be individualized in these cases [36].

### Ejection Fraction and New York Heart Association Functional Class

Investigations are ongoing as to whether earlier intervention with CRT in adults (NYHA class II) is beneficial. In children, considerations for earlier intervention may be important prior to adverse remodeling given the duration in which therapy will be required for heart failure and the higher relative incidence of transplant need when severe heart failure has its onset early in life [23].

### Chronic Right Ventricular Pacing

In adult patients who have drug refractory heart failure and ejection fraction less than 35% but are right ventricular paced (and as a result have a wide QRS), generally receive CRT therapy despite this situation not being specifically supported in randomized trials.

The issue is more complex when considering CRT in children. Should the implanter wait until severe cardiomyopathy develops? The answer to this question is presently undefined. Some studies suggest that children with complete AV block and constantly paced from the right ventricle developed cardiomyopathy, and this can be prevented or treated by upgrading their devices to include left ventricular pacing [33,37]. However, it remains unclear whether the right ventricular pacing alone produced cardiomyopathy or an underlying disease process was responsible or AV block and cardiomyopathy as well.

### Sinus Rhythm

The major randomized trials performed for CRT recruited adults in sinus rhythm. Whether or not a benefit exists for patients in atrial fibrillation or other atrial arrhythmias with CRT is being investigated. In children, the issues are even more complex. Cardiomyopathy in the setting of congenital heart disease is often complicated by scar-related atrial flutter that may be persistent in later stages. Clinical judgment must be individualized; however, in general, if ventricular rates can be well controlled (so as to allow biventricular pacing most of the time), then the atrial flutter alone should not preclude CRT recommendation.

Even when in sinus rhythm, children often have faster sinus rates and shorter atrioventricular conduction times. When sinus tachycardia is present, biventricular pacing that tracks the atrium may be rapid, and an element of tachycardia-related dysfunction may offset the benefits from resynchronization.

## Optimization of CRT Devices: Challenges in Children

Approximately a third of patients who receive CRT devices with appropriate and established indications for their placement do not respond to therapy. In certain cases, either the myocardial disease process is so far advanced or the substrate is worsening (new infarctions) that CRT benefit is far outweighed by these factors. In others, however, the left ventricular lead's position, function, and device programming may be suboptimal and result in the failure of benefit. A variety of techniques to try and optimize resynchronization devices in adults have been attempted, but none have yielded consistent and reproducible results.

### Echocardiographic Optimization

Since the expected result of CRT is mechanical resynchronization, echocardiographic parameters have been developed and tried to assess the extent of continuing dyssynchrony and then by reprogramming the device (offset between right and left ventricular stimulation) or repositioning the lead to check if optimal synchrony is occurring. A major issue with using these techniques in children is that of inadequate frame rates with presently existing systems. Conduction velocity, even in the diseased child's heart, is relatively rapid, and thus when frame rates are low, predicting the most dyssynchronous or latest to activate site can have unreliable spatial resolution.

The effects of exercise and posture may also be more relative in children than in adults. Despite relatively advanced ventricular dysfunction, children may be more active and prone to changing their position. In these circumstances, the intracardiac conduction velocities and pacing vectors may change appreciably (asymmetric decremental conduction), making any attempt at optimization at rest in the supine position unreliable. Children may also have substantial changes in their underlying substrate, particularly the population waiting for transplantation. New surgical scars, atrial arrhythmias, and the effects of conduction slowing membrane active medications once again will not be helped with attempts at optimization prior to these changes.

### Electrocardiographic Optimization

Since electrical stimulation must first occur prior to contraction of the myocardium, electrical synchrony has been used as a surrogate for mechanical synchrony, and attempts at using the 12-lead electrocardiogram to optimize CRT devices have arisen [3]. The QRS duration, fusion of electrocardiographic vectors, and recognition of anodal stimulation or inordinate capture latency have been used in adults, but their limitations must be understood when applying to the pediatric population [3].

#### QRS Duration

Normalization of the QRS duration with biventricular stimulation is an inexact but usable surrogate for synchronization. As noted above, in children, however, the QRS is often in the normal range (as compared to adults) even with advanced cardiomyopathy and anecdotally shown benefit with resynchronization.

#### QRS Vector Fusion

**Figure 8:**
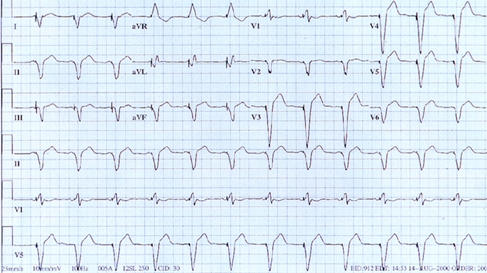
12-lead electrocardiogram with biventricular stimulation. Note, the right bundle-branch block pattern with negative QRS complex in lead I consistent with left ventricular stimulation. The negative QS waves in the inferior leads suggest either fusion with right ventricular pacing or placement of the left ventricular lead in a posterior and lateral location.

Each pacing site gives rise to a characteristic vector that can be deduced with analysis of the 12-lead electrocardiogram. For example, right ventricular pacing from the apex results in a left bundle-branch block pattern with negative QRS complexes in leads II, III, aVF, and a tall R wave in lead I. On the other hand, left ventricular pacing from the midlateral wall results in a right bundle-branch block pattern and a negative QRS in lead I. It follows, therefore, that biventricular stimulation when optimally resynchronizing the heart (equal contributions from both pacing sites), the electrocardiogram should be fused or intermediate between RV and LV pacing vectors (Figure 8). If, however, biventricular stimulation results in a pacing vector very similar to right ventricular pacing alone, then either the left ventricular lead has been placed too close to the right ventricle (anterior intraventricular vein) and should be repositioned or there is significant capture latency and exit delay from the pacing site to the rest of the ventricle. In the later situation, if recognized on the electrocardiogram, programming an offset so that the left ventricular lead output is delivered prior to the right ventricular lead output may better synchronize the heart and decrease symptoms.

Another cause for the electrocardiogram to appear like right ventricular pacing alone even with biventricular stimulation is the phenomenon of anodal stimulation [38]. This problem may be more frequent in children given the small size of their hearts. Here, despite the left ventricular pacing threshold being adequate when stimulated between the left ventricular lead tip and the RV lead (coil or ring electrode), the stimulation wave front is from the anode (RV lead) and thus negating any benefit from biventricular stimulation. The implanter should be aware of and look for this phenomenon with the electrocardiogram and if found, consider bipolar stimulation with the LV lead or programming the output or pacing vector differently.

Just as with adults, however, when CRT devices are placed in children, every effort to ensure close to 100% biventricular stimulation should be made. Causes that may be decreasing the amount of ventricular pacing including frequent PVCs, PACs that fall in the PVARP, atrial arrhythmias with rapid ventricular conduction, etc., should be sought and remedied.

## Conclusion

Cardiac resynchronization therapy has made a major impact in the lives of many adults with drug refractory congestive heart failure. While applying this therapy to children has been shown to be beneficial, distinct differences in how the physician should approach care in children exist. The standard indications often have to be modified with clinical judgment and techniques for optimization should be exactly understood prior to applying in the pediatric population.Specific difficulties associated with implanting a left ventricular pacing lead in children, and suggestions on how to overcome these difficulties have been reviewed in this paper.

## Figures and Tables

**Table 1:**
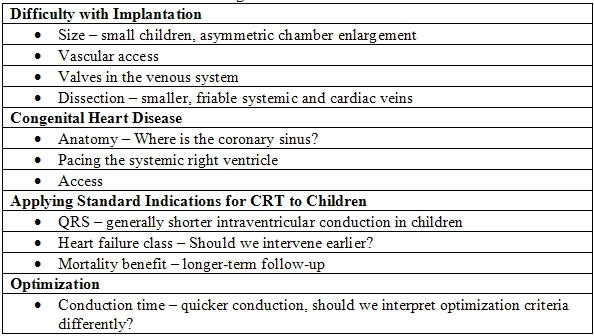
Challenges with CRT in Children

**Table 2:**